# Palmitoleic and Dihomo-γ-Linolenic Acids Are Positively Associated With Abdominal Obesity and Increased Metabolic Risk in Children

**DOI:** 10.3389/fped.2021.628496

**Published:** 2021-04-09

**Authors:** Man-Chin Hua, Hui-Min Su, Ming-Wei Lai, Tsung-Chieh Yao, Ming-Han Tsai, Sui-Ling Liao, Shen-Hao Lai, Jing-Long Huang

**Affiliations:** ^1^Department of Pediatrics, Chang Gung Memorial Hospital, Keelung, Taiwan; ^2^Chang Gung University College of Medicine, Taoyuan, Taiwan; ^3^Department of Physiology, National Taiwan University College of Medicine, Taipei, Taiwan; ^4^Division of Gastroenterology, Department of Pediatrics, Chang Gung Memorial Hospital, Taoyuan, Taiwan; ^5^Division of Allergy, Asthma and Rheumatology, Department of Pediatrics, Chang Gung Memorial Hospital, Taoyuan, Taiwan; ^6^Division of Chest, Department of Pediatrics, Chang Gung Memorial Hospital, Taoyuan, Taiwan; ^7^Department of Pediatrics, Municipal TuCheng Hospital, Chang Gung Memorial Hospital, New Taipei City, Taiwan

**Keywords:** abdominal obesity, children, dihomo-γ-linolenic acid, desaturase activities, metabolic risk, palmitoleic acid

## Abstract

**Background:** The impact of abdominal obesity (AO) on plasma fatty acid changes and cardiometabolic risk in children who are obese and overweight has rarely been investigated. This study determined whether plasma fatty acid composition differed between children with AO and those without AO and its relationship with metabolic risk, particularly in the obese and overweight groups.

**Methods:** A total of 181 schoolchildren (aged 7–18 years) were included. Anthropometric and biochemical data and plasma fatty acid profiles were analyzed, and the indices of desaturase activity were estimated. Children were categorized based on their body weight and AO status. A continuous metabolic risk score was calculated using the sum of the z-scores of metabolic variables. A one-way analysis of variance test was used to compare the composition ratio of fatty acids between children with and without AO in the obese and overweight groups and normal-weight controls. Pearson analysis was also used to explore significant fatty acid and desaturase indicators associated with metabolic abnormalities.

**Results:** Children who were obese and overweight (*N* = 126) displayed higher dihomo-γ-linolenic acid (20:3n-6) and γ-linolenic acid (18:3n-6) proportions than normal-weight controls (*N* = 55), but lower heptadecanoic acid (17:0) proportion, regardless of the AO status of each individual. Obese and overweight children with AO (*N* = 89), but not their non-AO counterparts (*N* = 37), exhibited a significantly higher proportion of palmitoleic acid (16:1n-7) than the remaining study groups. Pearson analysis showed that high proportions of palmitoleic acid and dihomo-γ-linolenic acid, as well as increased stearoyl-coenzyme A desaturase-1(16) and delta-6 desaturase and decreased delta-5 desaturase activities, are strongly correlated with weight-height ratio, homeostasis model of assessment values for insulin resistance, hypertriglyceridemia, and continuous metabolic risk scores.

**Conclusion:** Higher palmitoleic acid and dihomo-γ-linolenic acid proportions, as well as increased stearoyl-coenzyme A desaturase-1(16) and delta-6 desaturase and decreased delta-5 desaturase activities are associated with AO and increased metabolic risk in children who are obese and overweight.

## Introduction

Childhood obesity has been increasing at an alarming rate in recent decades (1). In obesity, hypertrophic adipocytes and adipose tissue-resident immune cells display a chronic proinflammatory profile by altering the secretion of adipokines and lipokines, which exacerbate cardiometabolic disease (2–4). Evidence also indicates that lipoprotein and fatty acid (FA) change in relation to abdominal obesity (AO) and overweight status (5, 6). In a birth cohort study, Kjellberg et al. reported that 26% of 6-year-old children already had one or more risk factors of metabolic syndrome, including increased waist circumference, dyslipidemia, insulin resistance (IR), and elevated blood pressure (7). Metabolic dysregulation during childhood has been shown to increase the risk of metabolic syndrome, type 2 diabetes, and cardiovascular diseases in adulthood (1, 8). Hence, it is crucial to detect and intervene in health threats from childhood.

Desaturase is involved in the biosynthesis of monounsaturated FA (MUFA) and polyunsaturated FA (PUFA) (9, 10). There is mounting evidence that changes in FA composition and desaturase activity are associated with metabolic diseases (11–14). Previous studies involving adults have identified the association between higher levels of palmitoleic acid (16:1n-7), dihomo-γ-linolenic acid (DGLA) (20:3n-6), oleic acid (18:1n-9), and γ-linolenic acid (18:3n-6), but a lower level of linoleic acid (18:2n-6); a higher stearoyl-coenzyme desaturase-1 (SCD1), delta-6 desaturase (D6D), but a lower delta-5 desaturase (D5D) activity for AO (11), metabolic syndrome (11, 15), and type 2 diabetes (13, 14). In pediatric populations, although a similar relationship was observed between changes in FA composition and hypertriglyceridemia (16) and higher cardiometabolic risk (17) in obese individuals (16, 18), whether children with AO display different FA characteristics and metabolic risk from children who are simply obese has rarely been investigated (19) and is of interest to physicians. It is reasonable to propose that AO, rather than weight status, may be a more important factor that influences plasma FA composition and metabolic risk in children.

In the present study, we first compared the differences in anthropometric, biochemical, and FA composition data in relation to body weight and AO status. Thereafter, we examined plasma FA profiles and how the indices of desaturase activity [SCD1(16), SCD1(18), D5D, and D6D] are associated with individual and combined metabolic risk factors. We aimed to investigate any significant association between plasma FA and metabolic risk factors in children who are obese and overweight, particularly among those with AO.

## Materials and Methods

### Study Participants

From March 2015 to October 2018, we recruited obese and overweight children aged 5–18 years who were willing to undergo anthropometric and body composition measurements from the outpatient clinic in Chang Gung Memorial Hospital, Keelung, in addition to normal-weight controls from the **P**rediction of **A**llergies in **T**aiwanese **Ch**ildren (PATCH) cohort study. Detailed information on demographic data and medical and general health conditions were obtained using questionnaires. Participants with chronic illness or receiving medications that could affect glucose or lipid metabolism were excluded from the study. This study was approved by the Ethics Committee of Chang Gung Memory Hospital (103-6519A3, 104-7100C, 106-3610C, and 201901820A3) and conducted in accordance with the Declaration of Helsinki as revised in 1983. Written informed consent was obtained from all children and their parents after they received detailed information on the objectives and design of the study.

### Anthropometric, Blood Pressure, and Body Composition Measurements

Each participant underwent comprehensive outpatient evaluation. All anthropometric indicators and body composition measurements were performed by two well-trained research assistants, following standardized international recommendations (12). Briefly, body weight (BW) and body height (BH) were measured using an electronic scale (Super-view-HW3050, Hualien, Taiwan; precision of 0.1 kg and 0.1 cm, respectively). BH and BW were used to calculate the body mass index (BMI) in kg/m^2^. Waist circumference was measured at the level of the umbilicus. Hip circumference was measured at the point of maximal buttock protrusion. Waist-to-hip (WHR) and waist-to-height (WHtR) ratios were calculated from these measurements. Blood pressure (BP) was measured using a vital signs digital monitor (Chang Gung Medical Technology, Taipei, Taiwan) on the upper right arm after a 10-min seated rest. Detailed body composition data, including body fat percentage, trunk fat percentage, total body fat, fat-free mass, total body water, and total body muscle (20) were obtained using a multi-frequency (20 and 100 kHz) bioelectrical impedance analysis device that uses an 8-point tactile electrode system (InBody 230, Seoul, Korea).

### Assessment of Dietary Intake

Starting April 2017, on the day of blood sampling and anthropometric measurements, a 1-month recall of the PATCH study-designed food frequency questionnaire (FFQ) was completed for each participant to estimate the usual frequency of consumption of the following food items: milk, fruits, vegetables, meat, shellfish, fish, nuts, juices, beverages, snacks, and fast foods. Each FFQ was filled with parental assistance. Each subject was asked how often they consumed individual food items using a 4-point scale (1: none; 2: 1–2 days per week; 3: 3–4 days per week; and 4: ≥5 days per week).

### Collection of Biochemical Data

Blood samples (10 mL) were collected from study participants after overnight fasting, and biochemical markers including glucose, insulin, triglyceride (TG), total cholesterol, low-density lipoprotein cholesterol (LDL-C), and high-density lipoprotein cholesterol (HDL-C) were determined using the electrochemiluminescence method (Roche cobas P512, Mannheim, Germany). The homeostasis model of assessment values for insulin resistance (HOMA-IR) was also obtained by using the formula proposed by Matthews et al.: insulin (μU/mL) × glucose (mg/dL)/405 (21).

### Plasma FA Analysis

Total lipids were extracted from the plasma samples and converted to their methyl ester equivalents as described previously (12, 22), and analyzed using an Agilent 7820A GC using flame ionization detection on a SP-2560 polar fused silica capillary column (100 m × 0.25 mm × 0.2 μm; Supelco Inc., PA, USA) with nitrogen as the carrier gas (12). The FA peaks were identified by comparing retention times with those of a standard mixture of GLC-68A, GLC-481B, GLC-532, GLC-744 (Nu-Chek Prep), 37 FAME, trans 16:1n-7, trans 18:1n-7, trans 18:1n-9, and cis/trans 18:2n-6 (all obtained from Supelco Inc., or Sigma). 13:0 free fatty acid (10 μg) was added as an internal standard. FA composition was expressed as the weight of a percentage of the total weight of carbon-12 to carbon-24 FAs (wt%). The estimated desaturase activity was calculated using the ratio of FA in plasma (11, 17, 23); SCD1(16) activity = (16:1n-7/16:0), SCD1(18) activity = (18:1n-9/18:0), D5D activity = (20:4n-6/20:3n-6), and D6D activity = (20:3n-6/18:2n-6).

### Definitions Used in This Study

**Abdominal obesity (AO) group:** Obese (BMI ≥ 95th percentile) or overweight (BMI ≥ 85–95th percentile) children (24) with WHtR ≥ 0.5 (25, 26).

**Non-AO group:** Obese or overweight children (24) with WHtR < 0.5.

**Normal-weight healthy controls (HC):** Normal-weight (BMI = 10–85th percentile) children (24) with a normal cardiometabolic profile, with WHtR < 0.5.

**Combined metabolic risk assessment:** Five risk factors were assessed in the study: (1) AO (WHtR ≥ 0.5) (25, 26); (2) high TG ≥ 150 mg/dL (27); (3) low HDL-C (boys < 40 mg/dL; girls < 50 mg/dL) (27); and (4) HOMA-IR ≥ 4.5 (currently, no universal definition of IR is applicable in children. Therefore, we used the 75th percentile of HOMA-IR of all participants as the threshold of IR); (5) hypertension: systolic or diastolic BP ≥ 95th percentile for children between 1 and 12 years of age, and BP ≥ 130/85 mmHg for 13-year-old and older individuals (28).

**Continuous metabolic risk (CMR) scores:** A CMR score was calculated using five metabolic variables, including WHtR, TG, HDL-C, HOMA-IR, and systolic BP. Each CMR score component was internally standardized using z-scores. The z-score values of each metabolic variable were then summed using the following equation: WHtR + TG – HDL-C + HOMA-IR + systolic BP. The z-score of HDL-C was multiplied by−1 because it was inversely associated with cardiometabolic risk.

### Statistical Analysis

Numerical variables were summarized as mean ± standard deviation (SD) or as frequencies and percentages. The normality of data distribution was checked. Associations between categorical variables were analyzed using the chi-square test. One-way analysis of variance (ANOVA) was used to test for differences in anthropometric and biochemical data, plasma FA composition, and estimated desaturase indices between the AO, non-AO, and HC groups. The correlation coefficients between selected FA proportions, estimated desaturase indices, obesity indices, metabolic risk factors, and CMR scores were determined using Pearson analyses. Furthermore, a multivariate regression analysis with a stepwise procedure adjusted for age, sex, and total saturated FA (SFA), MUFA, n3- and n6-PUFA concentrations, was performed to examine the associations between CMR scores, significant FA proportions, and indices of desaturase activity. Histograms were prepared using mean values and standard deviations. Statistical significance was set at *p* < 0.05. All statistical analyses were performed using IBM SPSS Statistics version 20 (Armonk, NY, USA).

## Results

### Study Participants

At the initial evaluation, 228 children ranging from 5 to 18 years of age were enrolled. To avoid potential confounding factors, we excluded 12 children with incomplete FA analysis, 34 normal-weight children with either WHtR ≥ 0.5 or cardiometabolic abnormalities, and one obese child with type 2 diabetes. Our final sample included 181 schoolchildren (96 males and 85 females, aged 7–18 years, with an average age of 15.01 ± 2.17 years). The characteristics of the children participating in this study are summarized in Table 1. In total, 99 (54.7%) study participants were obese, 27 (14.9%) were overweight, and 55 (30.4%) were normal-weight and served as healthy controls (HCs). Of the 126 children who were obese and overweight, 89 (70.6%) showed AO and the remaining 37 (29.4%) did not (Table 1). There were no intergroup differences in the mean age or sex. Based on the 1-month FFQ recall, the usual consumption of food items, including milk, fruits, vegetables, meat, shellfish, fish, nuts, juices, beverages, snacks, and fast foods did not differ significantly between the non-AO and AO groups (data not shown).

**Table 1:** Anthropometric and biochemical characteristics of the study participants.

	**Normal-weight healthy controls (HC) (*N* = 55)**	**Obese and overweight children (*****N*** **=** **126)**	
		**Non-abdominal obesity (non-AO group) (*N* = 37)**	**Abdominal obesity (AO group) (*N* = 89)**
**Basic and anthropometric data**			
Age (y)	15.67 ± 2.10	15.49 ± 1.80	14.44 ± 2.67
Gender, male (%)	31 (56.3)	23 (62.1)	41 (46.0)
BMI (kg/m^2^)	19.23 ± 1.75	26.58 ± 3.22^*^	27.33 ± 3.67^*^
Systolic BP (mmHg)	115.80 ± 11.88	126.59 ± 12.04^*^	127.79 ± 10.49^*^
Diastolic BP(mmHg)	70.35 ± 8.92	76.49 ± 10.14^*^	80.93 ± 8.06^*^^#^
Waist-hip ratio (WHR)	0.76 ± 0.05	0.81 ± 0.06^*^	0.93 ± 0.06^*^^#^
Waist-height ratio (WHtR)	0.42 ± 0.03	0.48 ± 0.03^*^	0.58 ± 0.06^*^^#^
**Data from bioelectrical impedance analysis**			
Body fat (%)	19.70 ± 7.64	32.04 ± 8.29^*^	38.80 ± 6.17^*^^#^
Trunk fat (%)	18.50 ± 8.79	33.44 ± 7.87^*^	39.31 ± 5.86^*^^#^
Total body fat (kg)	10.56 ± 5.41	23.75 ± 6.62^*^	25.77 ± 7.52^*^
Fat-free mass (kg)	41.08 ± 8.33	49.18 ± 10.63^*^	40.62 ± 10.56^#^
Total body water (kg)	30.14 ± 6.13	35.98 ± 7.76^*^	29.75 ± 7.70^#^
Total body muscle (kg)	22.43 ± 5.06	27.37 ± 6.47^*^	22.12 ± 6.33^#^
**Biochemistry data**			
Fasting blood glucose (mg/dL)	88.67 ± 5.22	90.04 ± 6.60	92.38 ± 25.45
Insulin (μlU/ml)	5.63 ± 8.23	11.12 ± 11.06^*^	17.34 ± 10.86^*^^#^
HOMA-IR	1.28 ± 0.72	2.37 ± 2.62^*^	4.68 ± 3.65^*^^#^
Triglycerides (mg/dL)	77.33 ± 71.13	105.16 ± 64.75^*^	113.04 ± 59.08^*^
Total cholesterol (mg/dL)	168.56 ± 34.97	172.50 ± 29.12	173.76 ± 30.77
HDL-C (mg/dL)	45.82 ± 14.66	47.56 ± 9.86	43.06 ± 8.65^#^
LDL-C (mg/dL)	93.11 ± 31.25	103.98 ± 29.43	108.15± 29.55^*^
**CMR scores (Z)**	−2.22 ± 2.61	0.45 ± 2.02^*^	1.61 ± 2.07^*^^#^

WHtR ≥ 0.5 was defined as abdominal obesity (AO). P-value based on ANOVA test with multiple comparison analysis.

* Indicates significant differences (P-values < 0.05) between the non-AO (or AO group) and HC group;

# *indicates significant differences (P-values < 0.05) between the AO and non-AO group in obesity/or overweight children*.

### Intergroup Comparison of Obesity Indices and Biochemical Data

As shown in Table 1, a significant and progressive increase in diastolic BP, WHR, WHtR, body fat percentage, and trunk fat percentage were observed in the HC, non-AO, and AO groups. Based on the metabolic profile, insulin, HOMA-IR, and CMR scores were significantly different among the three groups.

### Intergroup Comparison of Plasma FA Profile

We compared the plasma FA profiles of the HC, AO, and non-AO groups (Table 2). In general, neither total SFA nor MUFA or the n-6 or n-3 series of PUFAs showed any differences between the HC and obese children in the non-AO groups. Participants in the AO group exhibited a significantly higher proportion of MUFAs than the HC group (*P* = 0.001), and a lower proportion of n6-PUFA than the non-AO group (*P* = 0.048). Analysis of FA composition showed that children who were obese and overweight exhibited higher DGLA (20:3n-6), α-linolenic acid (18:3n-3), γ-linolenic acid (18:3n-6), docosapentaenoic acid (22:5n-6), and clupanodonic acid (22: 5n-3) proportions, whereas lower heptadecanoic acid (17:0) and stearic acid (18:0) proportions than those in the normal-weight controls, regardless of whether participants had AO. Participants in the AO group exhibited higher proportions of palmitoleic acid (16:1n-7), DGLA (20:3n-6), and docosapentaenoic acid (22:5n-6), but lower proportions of myristoleic acid (14:1), vaccenic acid (18:1n-7), linoleic acid (18:2n-6), and nervonic acid (24:1n-9) than did participants in the non-AO group. The proportions of other FAs between the study groups are shown in Table 2.

**Table 2:** Plasma fatty acid profile and estimated desaturase activities with regard to body weight status and abdominal obesity.

	**Normal-weight healthy controls (HC) (*N* = 55)**	**Obese and overweight children (*****N*** **=** **126)**	
		**Non-abdominal obesity (non-AO group) (*N* = 37)**	**Abdominal obesity (AO group) (*N* = 89)**
**Plasma fatty acid composition**	**Mean (wt%)** **±** **SD**	**Mean (wt%)** **±** **SD**	**Mean (wt%)** **±** **SD**
**SFA**	34.73 ± 4.38	33.52 ± 2.30	33.97 ± 2.64
14:0	0.52 ± 0.22	0.60 ± 0.28	0.66 ± 0.24^*^
16:0	23.05 ± 1.52	22.93 ± 1.74	23.27 ± 1.61
17:0	0.25 ± 0.09	0.20 ± 0.05^*^	0.18 ± 0.05^*^
18:0	9.08 ± 2.87	8.07 ± 1.33^*^	8.19 ± 1.59^*^
20:0	0.30 ± 0.09	0.27± 0.06	0.26 ± 0.06^*^
22:0	0.86 ± 0.23	0.82 ± 0.20	0.78 ± 0.18^*^
24:0	0.66 ± 0.24	0.62 ± 0.19	0.62 ± 0.20
**MUFA**	20.07 ± 2.25	20.74 ± 2.78	21.51± 2.30^*^
14:1	0.10 ± 0.07	0.08 ± 0.05	0.05 ± 0.04^*^^*^
16:1n-7	1.32 ± 0.42	1.48 ± 0.53	1.85 ± 0.55^*^^*^
18:1n-9	15.70 ± 1.91	16.44 ± 2.52	17.17 ± 2.06^*^
18:1n-7	1.40 ± 0.21	1.32 ± 0.20	1.23 ± 0.21^*^^*^
20:1n-9	0.10 ± 0.03	0.11 ± 0.08	0.10 ± 0.04
24:1n-9	1.46 ± 0.43	1.31 ± 0.48	1.11 ± 0.29^*^^*^
**n-6 PUFA**	41.90 ± 4.04	42.13 ± 3.55	40.72 ± 3.39^*^
18:2n-6	34.09 ± 3.90	33.74 ± 3.47	32.14 ± 3.56^*^^*^
18:3n-6	0.19 ± 0.19	0.30 ± 0.20^*^	0.34 ± 0.21^*^
20:3n-6	1.08 ± 0.27	1.32 ± 0.36^*^	1.56 ± 0.36^*^^*^
20:4n-6	6.17 ± 1.22	6.42 ± 1.69	6.28 ± 1.05
22:4n-6	0.24 ± 0.15	0.19 ± 0.05^*^	0.22 ± 0.06
22:5n-6	0.13 ± 0.05	0.15 ± 0.05^*^	0.18 ± 0.05^*^^*^
**n3-PUFA**	3.61 ± 0.77	3.88 ± 1.01	3.82 ± 0.90
18:3n-3	0.49 ± 0.13	0.59 ± 0.19^*^	0.60 ± 0.18^*^
20:5n-3	0.61 ± 0.27	0.65 ± 0.30	0.56 ± 0.29
22:5n-3	0.35 ± 0.09	0.40 ± 0.12^*^	0.39 ± 0.10^*^
22:6n-3	2.17 ± 0.52	2.24 ± 0.78	2.27 ± 0.61
**Desaturase index**			
SCD1_(16)_	0.06 ± 0.02	0.06 ± 0.02	0.08 ± 0.02^*^^*^
SCD1_(18)_	1.86 ± 0.49	2.09 ± 0.47^*^	2.15 ± 0.40^*^
D5D	5.94 ± 1.47	5.28± 2.18^*^	4.22 ± 1.17^*^^*^
D6D	0.03± 0.01	0.04 ± 0.01^*^	0.05 ± 0.01^*^^*^
**Plasma fatty acid concentrations** (μg/ml)	2,396.97 ± 547.22	2,795.63 ± 947.49^*^	2,803.31 ± 853.72^*^
SFA (μg/ml)	835.29 ± 234.66	944.25 ± 377.34	956.31 ± 312.51^*^
MUFA (μg/ml)	480.86 ± 124.85	591.51 ± 259.12^*^	606.71 ± 219.07^*^
n6-PUFA (μg/ml)	1,000.44 ± 230.72	1,157.99 ± 301.41^*^	1,132.86 ± 323.32^*^
n3-PUFA (μg/ml)	87.32 ± 29.79	108.10 ± 45.38^*^	107.65 ± 42.07^*^

WHtR ≥ 0.5 was defined as abdominal obesity (AO). P-value based on ANOVA test with multiple comparison analysis.

* Indicates significant differences (P-values < 0.05) between the non-AO (or AO group) and HC group;

# *indicates significant differences (P-values < 0.05) between the AO and non-AO group in obesity/or overweight children. SFA, saturated fatty acid; MUFA, monounsaturated fatty acid; PUFA, polyunsaturated fatty acid*.

We also compared the plasma FA concentrations in the HC, AO, and non-AO groups (Table 2). Our data revealed that children who were obese and overweight, regardless of AO status, exhibited significantly higher levels of total plasma FAs (μg/mL), as well as MUFA (μg/mL), n6-polyunsaturated FA (PUFA) (μg/mL), and n3-PUFA concentrations (μg/mL) than the HC group.

### Intergroup Comparison of Desaturase Activities

The estimated levels of desaturase activity are presented in Table 2. Participants in the AO group displayed significantly higher SCD1(16) and D6D activities, but lower D5D activity, than the HC and non-AO groups (all *P* < 0.05). Nonetheless, the SCD1(18) activity was higher in the children who were obese and overweight but showed no differences between the AO and non-AO groups (*P* > 0.05) (Table 2).

### Correlation Between FA Compositions, Desaturase Activities, and Cardio-Metabolic Risk Markers

We detected positive correlations between metabolic variables (insulin, HOMA-IR, and TG levels), CMR scores, and anthropometric indices (BMI, WHR, WHtR, and trunk fat percentage) and the proportions of palmitoleic acid (16:1n-7) (*r* = 0.41–0.51), DGLA (20:3n-6) (*r* = 0.22–0.55), γ-linolenic (18:3n-6) (*r* = 0.24–0.35), and activity of SCD1(16) (*r* = 0.38–0.50) and D6D (*r* = 0.27–0.53); an inverse correlation with heptadecanoic acid (C17:0) (*r* = −0.31 to −0.52) and D5D expression (*r* = −0.42 to −0.51) was also observed (Table 3). Nonetheless, these parameters were weakly correlated with SCD1(18) activity (*r* = 0.20–0.55). Associations of systolic BP, glucose, total cholesterol, and HDL-C are shown in Table 3. In the stepwise logistic regression model adjusted for age, sex, and total SFA, MUFA, n3- and n6-PUFA concentrations, D5D activity was selected as an independent indicator inversely associated with CMR scores (adjusted beta = −0.21, *P* = 0.019).

**Table 3:** Correlation coefficient between single fatty acid proportions, estimated desaturase activities, and selected metabolic risk factors.

	**16:1n-7 (wt%)**	**20:3n-6 (wt%)**	**17:0 (wt%)**	**18:3n-6 (wt%)**	**SCD1(16)**	**SCD1(18)**	**D5D**	**D6D**
**Anthropometric data**								
BMI (kg/m^2^)	0.41^***^	0.54^***^	−0.44^***^	0.28^***^	0.40^**^	0.26^**^	−0.50^***^	0.50^***^
Systolic BP (mmHg)	0.29^***^	0.31^***^	−0.24^***^	0.29^***^	0.27^**^	0.25^**^	−0.33^***^	0.28^**^
Waist-hip ratio	0.39^**^	0.52^***^	−0.47^***^	0.30^***^	0.38^***^	0.20^**^	−0.51^***^	0.49^***^
Waist-height ratio	0.47^***^	0.55^***^	−0.52^***^	0.33^***^	0.47^***^	0.23^**^	−0.51^***^	0.53^***^
Trunk fat (%)	0.50^***^	0.50^***^	−0.51^***^	0.30^***^	0.50^***^	0.20^**^	−0.45^***^	0.48^***^
**Metabolic profiles**								
Fasting blood glucose (mg/dL)	NS	NS	NS	NS	NS	NS	NS	NS
Insulin (μlU/ml)	0.44^***^	0.48^***^	−0.49^***^	0.35^***^	0.41^***^	0.26^**^	−0.46^***^	0.48^***^
HOMA-IR	0.45^***^	0.43^***^	−0.42^***^	0.34^**^	0.42^***^	0.37^**^	−0.49^***^	0.44^***^
Triglycerides(mg/dL)	0.51^***^	0.22^**^	−0.31^***^	0.24^**^	0.44^***^	0.55^***^	−0.42^***^	0.27^***^
Total cholesterol (mg/dL)	0.26^***^	0.23^***^	−0.17^*^	NS	0.25^**^	0.15^*^	−0.17^*^	0.22^**^
HDL-C (mg/dL)	NS	0.22^*^	NS	NS	NS	NS	NS	0.21^*^
**CMR scores**	0.41^***^	0.35^***^	−0.46^***^	0.27^**^	0.38^***^	0.36^***^	−0.43^***^	0.37^***^

Results based on Pearson correlation analysis. Significance levels:

* *P < 0.05*,

** *P < 0.01*,

*** *P < 0.001, NS: P > 0.05*.

Furthermore, our data revealed that WHtR presented a stronger correlation with trunk fat percentage (*r* = 0.76 vs. 0.71), insulin (*r* = 0.62 vs. 0.58), triglyceride (*r* = 0.34 vs. 0.31) levels, and total plasma FA concentrations (*r* = 0.21 vs. 0.16) than with BMI (data not shown).

### Association Between FA Compositions, Desaturase Activities, and Estimated Metabolic Risk

**Figure 1:**
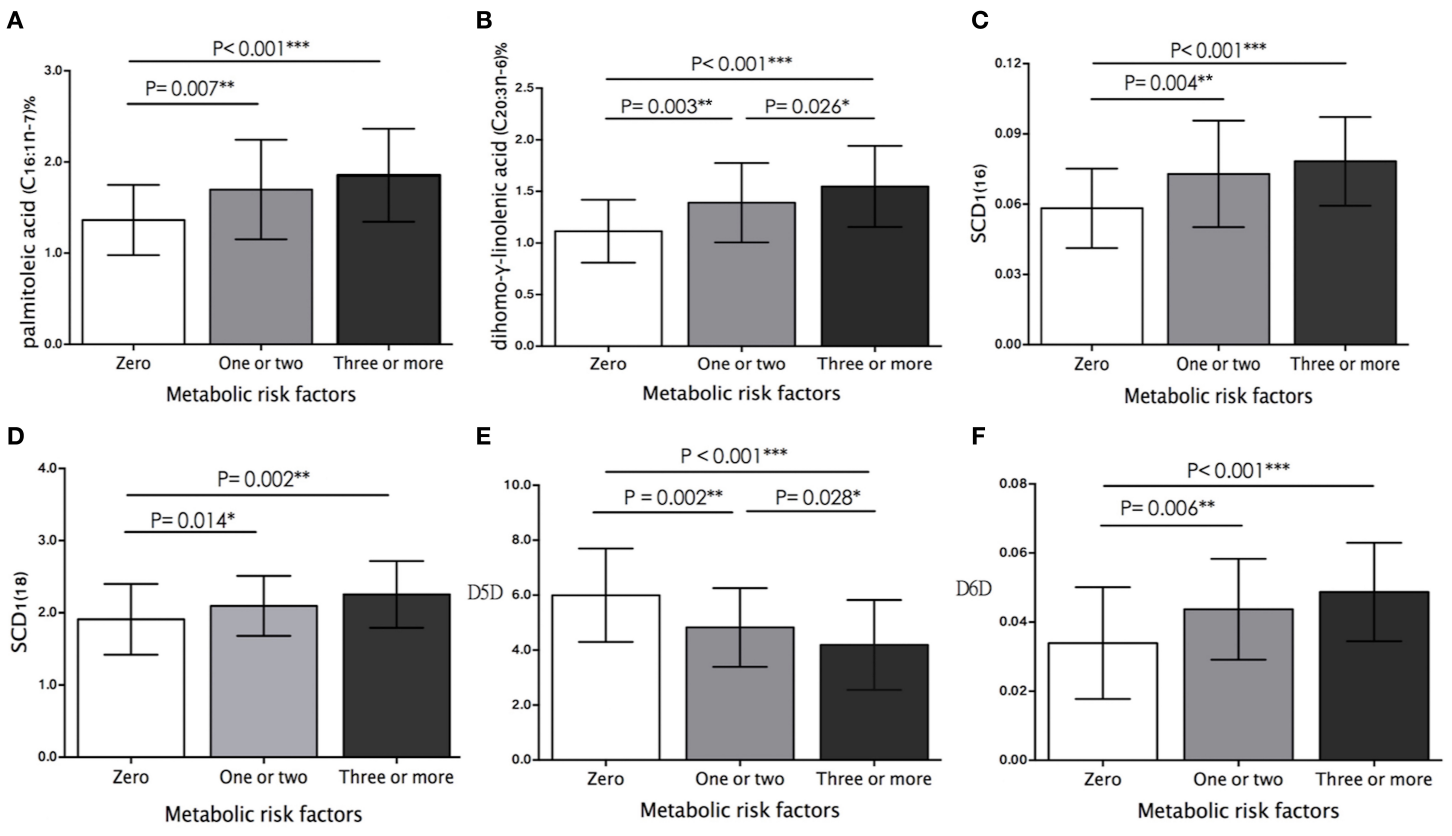
Comparison of plasma **(A)** palmitoleic acid proportion, **(B)** dihomo-γ-linolenic acid proportion (DGLA), and estimated activity of **(C)** SCD1(16), **(D)** SCD1(18), **(E)** D5D, and **(F)** D6D between study participants who had zero, one or two, and more than three metabolic risk factors. Histograms were prepared using mean values and SD. The differences between study groups are based on ANOVA test with multiple comparison analysis. Significance levels: *P < 0.05, **P < 0.01, ***P < 0.001.

Hypertension was observed in 90 (49.7%), AO in 89 (49.2%), low HDL-C in 88 (48.6%), IR in 45 (24.9%), and hypertriglyceridemia in 28 (15.5%) children. Figure 1 shows the metabolic risk clustering based on the assessment of the above-mentioned five risk factors. In total, 51 (28.2%) children had three or more risk factors, 75 (41.4%) had one or two risk factors, and 55 (30.4%) had none of the risk factors. Likewise, the proportions of palmitoleic acid (16:1n-7) and DGLA (20:3n-6) (Figures 1A,B), and the activities of SCD1(16), SCD1(18), and D6D (Figures 1C,D,F) showed an upward trend, whereas the activity of D5D showed a downward trend as metabolic alterations increased (Figure 1E). Of note, we observed that the proportion of DGLA (20:3n-6) (1.08 ± 0.27% vs. 1.39 ± 0.38% vs. 1.55 ± 0.39%, *P* < 0.001) and D5D activity (5.94 ± 1.47 vs. 4.82 ± 1.23 vs. 4.19 ± 1.63, *P* < 0.001) changed significantly from zero, to one or two, in the ≥ 3 metabolic risk factors groups (Figures 1B,E).

## Discussion

In the present study, we first explored the impact of obesity on plasma FA changes and metabolic risk in children. The results showed that higher DGLA (20:3n-6) and γ-linolenic acid (18:3n-6) proportions, but a lower proportion of heptadecanoic acid (17:0) was observed in children who were obese and overweight than in normal-weight healthy controls. In addition, the proportions of DGLA (20:3n-6) and γ-linolenic acid (18:3n-6) showed a positive correlation, whereas the proportion of heptadecanoic acid (17:0) was inversely correlated with IR, hypertriglyceridemia, and CMR scores. Taken together, our data suggest that DGLA (20:3n-6) and γ-linolenic acid (18:3n-6) proportions tended to increase, but the proportion of heptadecanoic acid (17:0) tended to decrease in children who were either obese or overweight with increased metabolic risk. Adipose tissue is an endocrine organ secreting factor that can both improve and impair insulin sensitivity (4). In obesity, elevated FA levels are associated with reduced glucose uptake by peripheral tissues and increased IR (4). Furthermore, elevated FA levels may drive lipid accumulation in visceral adipose tissue and very-low-density lipoprotein triglyceride synthesis and release, contributing to the dyslipidemic phenotype of metabolic syndrome (2–4). Consistent with our findings, the literature reveals that higher proportions of DGLA (20:3n-6) (11, 13, 15, 18, 29) and γ-linolenic acid (18:3n-6) (11, 13, 29) in plasma are linked to obesity and metabolic syndrome components. Heptadecanoic acid (17:0), a minor SFA in ruminant fat, has been reported to be an objective biomarker of milk fat intake (30). Warensjö et al. reported a relationship between heptadecanoic acid (17:0) and a lower risk of myocardial infarction in women (30). Sawh et al. also reported that plasma iso- C17:0 was inversely correlated with hepatic steatosis in children (31). More studies are required to validate the potential metabolic benefits of heptadecanoic acid in cardiometabolic health in humans (32, 33).

Next, we explored the impact of AO on plasma FA changes and metabolic risk in children. In comparison to peripheral fat, the accumulation of intra-abdominal fat has been strongly associated with metabolic disorders (34). A WHtR ≥ 0.5 has been reported as an appropriate cut-off point for classifying cardio-metabolic risk in children and adolescents, regardless of sex, age, and ethnicity (22, 35–37). Although we observed a significant and progressive increase in DGLA (20:3n-6) proportions from the HC to the non-AO and AO groups, whether abdominal or body fat made a greater contribution to the result was uncertain. Our data revealed that children who were obese and overweight and with AO, but not their non-AO counterparts, exhibited a significantly higher proportion of palmitoleic acid (16:1n-7) than the remaining study groups. Furthermore, we demonstrated that elevated palmitoleic acid (16:1n-7) proportion, in conjunction with increased SCD1(16) activity, are positively associated with IR, hypertriglyceridemia, and CMR scores. Notably, palmitoleic acid (16:1n-7) is exclusively regulated by SCD1(16) activity and is regarded as an important product of *de novo* lipogenesis (38). Our findings imply that increased palmitoleic acid (16:1n-7) proportion, a product related to SCD1(16) activity, not only increases the likelihood of abdominal fat accumulation, but also obesity-related metabolic abnormalities. The question of whether elevated palmitoleic acid (16:1n-7) has adverse effects on metabolic disorders remains controversial (9). Our findings are consistent with those of other reports (15, 18, 23, 38). Nonetheless, several animal and human studies have reported that palmitoleic acid (16:1n-7) may enhance whole-body insulin sensitivity, increase hepatic FA oxidation, and improve blood lipid profile (39), and are associated with a low prevalence of diabetes and cardiovascular risk (9, 40, 41). The discrepancies in the effects of palmitoleic acid (16:1n-7) on insulin actions and metabolic outcomes may be explained by the different ethnicities, ages, underlying health conditions, and the duration of such conditions (9).

Finally, consistent with previous reports, the present study showed that high D6D (16–18) and low D5D (11, 15, 18, 19) activities were significantly associated with metabolic risk. Furthermore, D5D was the best indicator of CMR scores in our study. D6D and D5D are critical for PUFA metabolism, Linoleic acid (18:2n-6) is metabolized to γ-linolenic acid (18:3n-6) by D6D, and then progressively elongated to DGLA (20:3n-6). D5D is the rate-limiting enzyme that converts DGLA to arachidonic acid (20:4n-6) (42, 43). Previous studies have revealed that single nucleotide polymorphisms located within or near the FADS1- FADS2 gene cluster on chromosome 11 may alter desaturation rates of both D6D and D5D, as well as the associated PUFA levels (44, 45). Minor allele carriers of rs174546, rs174547, and rs174566 were identified to be associated with increased DGLA, reduced arachidonic acid levels, and lower D5D activity (44–46). In addition, rs968567 was linked to higher D6D activity (45). Further longitudinal studies to explore the genetic variability associated with PUFA metabolism for those individuals suffering metabolic abnormalities since childhood would be potentially interesting.

The strength of this study lies in the distinct comparison of plasma FA profiles from all lipid classes and cardiometabolic risk between children with and without AO in the obese and overweight groups, and normal-weight, metabolic healthy controls. To date, several pediatric studies have reported that plasma FA or desaturase activity changes might be linked to obesity (16, 18, 38), whereas some studies have provided limited FA profiles (19, 38, 47), desaturase indicators (16), data obtained from different plasma components (15, 18), or based on the context of Western countries (16, 17). Only a few pediatric studies have investigated the impact of AO on plasma FA changes (19). However, this was a small sample size study (*n* = 58) that classified AO and non-AO participants by IDFEFICS criteria released for European children (48), and both obese and non-obese children with AO were included in the analysis, which likely led to mixed results (19). In another pediatric study, Choi et al. suggested that waist circumference and D6D levels could predict future IR and metabolic risk in Korean boys; whether WHtR had similar associations was not confirmed in the study (18).

The present study has some limitations. First, the cross-sectional design could not be used to determine causality. Next, although a 1-month recall of FFQ was applied to evaluate the dietary intake and the usual consumption of food frequency in the 1-month recall questionnaires, we did not access detailed information on total food fat, carbohydrate, energy, or habitual dietary intake in the study. Therefore, we were unable to adjust for these important covariates in the logistic regression analysis. Evidence indicates that carbohydrate and fat consumption may influence FA composition and SCD1 and D6D activities (49, 50). Furthermore, it is notable that the composition and incorporation of FA in the plasma and blood cells are the result of distinct processes, such as short- or long-term dietary intake, metabolism, and peripheral utilization (51). This study only measured plasma FA reflecting intake status and *de novo* lipogenesis over days to weeks; FA in other blood cells reflecting long-term effects was not measured; therefore, the present results on FA composition and its association with metabolic risk may differ from other sources of analyzed samples (51). Finally, although the age and sex distributions were similar between the AO and non-AO groups, we did not evaluate the hormonal changes and puberty stages in our study participants, which may influence body fat distribution and FA profiles (52). Therefore, our data should be interpreted with caution.

In summary, our data suggest that higher DGLA (20:3n-6) and γ-linolenic acid (18:3n-6), but lower heptadecanoic acid (17:0) proportions were more prevalent in children who are obese and overweight with increased metabolic risk. Furthermore, increased palmitoleic acid (16:1n-7) proportion, a product related to SCD1(16) activity, is associated with abdominal fat accumulation and obesity-related metabolic abnormalities. Overall, in our study, low D5D activity was the best indicator of CMR scores.

## Data Availability Statement

The datasets generated for this study are available upon reasonable request from the corresponding author.

## Ethics Statement

The studies involving human participants were reviewed and approved by Research Ethics Committee of Chang Gung Memory Hospital (103-6519A3, 104-7100C, 106-3610C, and 201901820A3). Written informed consent to participate in this study was provided by the participants' legal guardian/next of kin.

## Author Contributions

M-CH and H-MS involved in the laboratory work, statistical analysis and interpretation of its results. M-CH wrote the first draft of the manuscript, and J-LH and H-MS edited it. All authors were involved in the study design, recruitment of participants, written consent, reviewed the manuscript and approved the final version of the manuscript.

## Conflict of Interest

The authors declare that the research was conducted in the absence of any commercial or financial relationships that could be construed as a potential conflict of interest.

## Abbreviations

AO: abdominal obesity
CMR scores: continuous metabolic risk scores
DGLA: dihomo-γ-linolenic acid
D5D: Delta-5 desaturase
D6D: Delta-6 desaturase
FA: fatty acids
FFQ: food frequency questionnaire
MUFA: monounsaturated fatty acids
PUFA: polyunsaturated fatty acids
SCD1: stearoyl-coenzyme desaturase-1
SFA: saturated fatty acid
TG: triglyceride
WHR: waist-to-hip ratio
WHtR: waist-to height ratio.
